# A case of Epstein-Barr virus acute retinal necrosis successfully treated with foscarnet

**DOI:** 10.1016/j.ajoc.2022.101363

**Published:** 2022-01-29

**Authors:** Kayo Suzuki, Kenichi Namba, Keitaro Hase, Kazuomi Mizuuchi, Daiju Iwata, Takako Ito, Nobuyoshi Kitaichi, Hiroshi Takase, Susumu Ishida

**Affiliations:** a Department of Ophthalmology, Faculty of Medicine and Graduate School of Medicine, Hokkaido University, Sapporo, Japan; b Department of Ophthalmology, Health Sciences University of Hokkaido, Sapporo, Japan; c Department of Ophthalmology & Visual Science, Graduate School of Medical and Dental Sciences, Tokyo Medical and Dental University, Tokyo, Japan

**Keywords:** Acute retinal necrosis, Epstein-Barr virus, Polymerase chain reaction, Acyclovir, Ganciclovir, Foscarnet, ARN, acute retinal necrosis, VZV, varicella-zoster virus, HSV, herpes simplex virus, EBV, Epstein-Barr virus, EBV-ARN, ARN caused by EBV, CMV, cytomegalovirus, PCR, polymerase chain reaction

## Abstract

**Purpose:**

Epstein-Barr virus (EBV) is a herpes virus known to cause infectious mononucleosis and several other human disorders. Ocular EBV infections that have been reported include uveitis, retinal vasculitis, and acute retinal necrosis (ARN). ARN is usually caused by herpes simplex virus (HSV) or varicella-zoster virus (VZV). ARN that is caused by EBV (EBV-ARN) is rarely seen, and only a few cases have been reported. The visual prognosis for EBV-ARN is poor, and no treatment strategy has been established. We report on a patient who was treated successfully for EBV-ARN.

**Observation:**

An 80-year-old female who had been treated with prednisolone at 5 mg/day and methotrexate at 2 mg/week for rheumatoid arthritis visited our hospital because of blurred vision in her left eye. Her left visual acuity was 20/50, and extensive white-yellowish retinal lesions at the temporal periphery with retinal hemorrhages were seen through vitreous haze. The DNA sequence of EBV, but not of HSV, VZV, or cytomegalovirus, was detected by a polymerase chain reaction (PCR) assay in the aqueous humor (4.2 × 10^6^ copies/ml), with EBV also being positive in serum (3.5 × 10^2^ copies/ml). The patient received 2 mg of intravitreal ganciclovir injections twice with a 3-day interval and intravenous infusion of acyclovir at 750 mg/day for 7 days; however, the retinal white lesions expanded rapidly, then dose of prednisolone was increased (40 mg/day) and vitrectomy was performed 10 days after the initial visit. After the surgery, the retinal lesion continued to enlarge. Vitreous samples showed high copies of EBV (1.2 × 10^8^ copies/ml). Following treatment with intravenous foscarnet (4800 mg/day), which replaced the acyclovir application, the retinal white lesions gradually diminished, leaving retinal scars. To date, the patient has developed no retinal detachment and shows visual acuity over 6/60 in the left eye along with silicone oil.

**Conclusions:**

We experienced a case of EBV-ARN that was refractory to systemic acyclovir and topical ganciclovir but responded effectively to systemic foscarnet after vitrectomy. Although the clinical management remains challenging in this disease, foscarnet is considered to be one of the candidate drugs for EBV infections.

## 1 Introduction

Acute retinal necrosis (ARN) is a sight-threatening necrotizing retinitis and commonly develops in immunocompetent hosts. The most common cause of ARN is the herpes virus family, particularly varicella-zoster virus (VZV) and herpes simplex virus (HSV) type 1 and type 2.^1^^,^^2^ However, some ARN cases in which Epstein-Barr virus (EBV) was highly suspected as the cause of disease have recently been reported,^3^, ^4^, ^5^, ^6^, ^7^, ^8^, ^9^, ^10^, ^11^ and the visual prognoses of all of these were poor. We report a case of ARN caused by EBV (EBV-ARN) that was successfully treated with foscarnet.

## 2 Case report

**Fig. 1:**
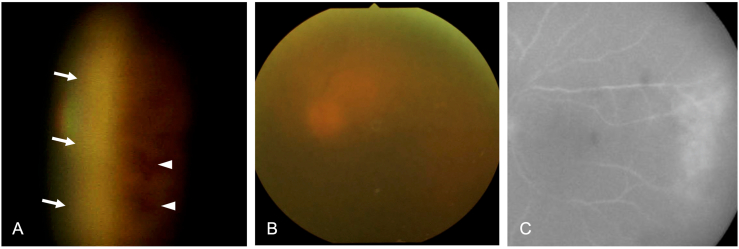
Fundus photographs and fluorescein angiography findings of the left eye at the initial visit **A.** Temporal retina of the left eye showed well-demarcated creamy area (arrow) and retinal hemorrhages (arrowhead) at temporal periphery with slit-lamp fundus lens (inverted image). **B.** Fundus photography of the left eye showed redness of optic disc through grade 1+ vitreous haze. **C.** Fluorescein fundus angiography of the left eye showed dye leakage from retinal vessels and staining of temporal area corresponding to white lesions.

**Fig. 2:**
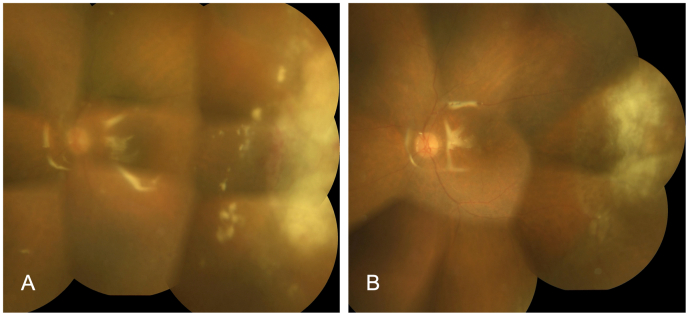
Fundus photographs of the left eye after vitrectomy **A.** Eleven days after vitrectomy, white-yellowish retinal lesions with retinal hemorrhages at temporal periphery continued to be enlarged. **B.** Twenty-five days after vitrectomy, retinal white lesions were gradually diminished leaving retinal scars.

An 80-year-old female suddenly noticed blurred vision in her left eye and visited our hospital. She had been treated with 5 mg/day of prednisolone and 2 mg/week of methotrexate for rheumatoid arthritis for more than 2 years. At the first visit, her visual acuity was 20/50 in the left eye. The intraocular pressure was 6 mmHg in the left eye. Slit-lamp examination revealed the presence of inflammation in the anterior chamber (1+ flare and 1+ cells) with mutton-fat keratic precipitates and mild senile cataracts; however, no iris atrophy or peripheral anterior synechia were seen. Fundus examination of the left eye showed 1+ vitreous haze, redness and swelling of the optic disc, and extensive white-yellowish retinal lesions at the temporal periphery with retinal hemorrhages (Fig. 1A and B). The patient's right eye was within normal limits without any inflammatory findings. Fluorescein angiography of the left eye demonstrated dye leakage from the retinal vessels and staining of the temporal area corresponded to the white lesions (Fig. 1C). Examination of the right eye was unremarkable. Hematologic workup revealed that the white blood cell count was normal: 4700/μl (normal ranges: 3300–8600/μl), the C-reactive protein was high: 2.28 mg/dl (normal ranges: <0.14 mg/dl), and the soluble interleukin-2 receptor was high: 966 U/ml (normal ranges: ≤ 459 U/ml). The cytomegalovirus (CMV) antigenemia test was positive, with pp65 antigen cells (C7-HRP: 6/50,000 cells). The EBV antibody test was negative for IgG against early antigens, but positive for IgG against viral capsid antigens and nuclear antigens. Then the anterior chamber was tapped, and DNA was extracted from the aqueous humor. The DNA sequence of EBV was detected by polymerase chain reaction (PCR) assay in the aqueous humor (4.2 × 10^6^ copies/ml), however sequences of HSV, VZV, or CMV were never detected. The DNA of EBV was also positive in serum (3.5 × 10^2^ copies/ml). Based on these results, EBV-ARN was suspected. The patient received 2 mg of intravitreal ganciclovir injections twice with a 3-day interval and intravenous infusion of acyclovir at 750 mg/day for 7 days; however, the vitreous haze increased to grade 3+, and the retinal white lesions expanded rapidly. Ten days after the initial visit, the patient underwent a 25-gauge pars plana vitrectomy, lensectomy, intraocular lens implant, scleral buckle placement, and laser photocoagulation and silicone oil tamponade, and dose of prednisolone was increased from 5 mg/day to 40 mg/day. After the vitrectomy, the vitreous haze disappeared; however, the retinal white lesion continued to enlarge (Fig. 2A). Vitreous fluid samples by PCR assay showed high copies of EBV-DNA (1.2 × 10^8^ copies/ml) but not of HSV, VZV, CMV, or toxoplasma gondii. Histological examination showed the cell block of the vitreous fluid sample^12^ to be positive for EBV-encoded RNA by in situ hybridization, but negative for CMV-positive cells or bacterial cells. We diagnosed the patient with EBV-ARN and considered the case as being acyclovir-resistant. Three days after the surgery, intravenous infusion of foscarnet (twice a day at 50 mg/kg each; 4800 mg/day), which replaced the acyclovir treatment, was administered to the patient. After starting the treatment, the retinal white lesions gradually diminished leaving retinal scarring (Fig. 2B). Retinal detachment did not develop with silicone oil tamponade, and the visual acuity has been maintained at above 6/60 in the left eye.

## 3 Discussion

Diagnosis of ARN is based on the ophthalmic findings of retinal necrosis with discrete borders located in the peripheral retina, together with rapid, circumferential progression of necrosis, evidence of occlusive vasculitis and arterial involvement, and prominent inflammatory reaction in the vitreous and anterior chamber.^13^ The ophthalmic findings of the present case were consistent with these characteristic ocular findings for ARN.^13^, ^14^, ^15^

In this case, there were high copy numbers of EBV-DNA in the aqueous humor (4.2 × 10^6^ copies/ml) and vitreous humor (1.2 × 10^8^ copies/ml), higher than that in serum (3.5 × 10^2^ copies/ml). In addition, no other possible pathogens of ARN, namely, HSV, VZV, CMV, or toxoplasma, were detected by PCR assay in the aqueous humor or the vitreous humor. The histological detection of EBV-encoded RNA in the vitreous also supports the conclusion that the pathogen was EBV. Based on these results, we considered that the pathogen of this ARN case is EBV.

Several papers reported that EBV can be the sole pathogen of necrotizing retinitis (Table 1).^3^, ^4^, ^5^, ^6^, ^7^, ^8^^,^^10^ Ocular findings in some cases showed severe necrotizing lesions in peripheral areas that are very similar to the findings seen in HSV- ARN and VZV-ARN.^3^, ^4^, ^5^^,^^7^^,^^8^^,^^10^ However, in some reports showed the necrotizing lesions were located in the peripapillary area,^6^^,^^9^^,^^11^ which are atypical in HSV-ARN and VZV-ARN. Regardless of the locations of the necrotizing lesions, all the cases showed rapid and circumferential progression of the necrotizing retinitis and resulted in poor visual prognoses.

**Table 1:** Case reports on EBV-related necrotizing retinitis.

Author (year of publication)	Age/sex	Laterality	Comorbidity (Treatment)	Method of diagnosis	Antiviral systemic therapy (Intravitreal injection)	Visual prognosis
Grossniklaus (1993)^3^	6/M	Bilateral	XLP	EBV-DNA Positive in enucleated eye	NA	NLP (OD) 20/50 (OS)
Kramer (2001)^4^	32/F	Unilateral	NA	Serological EBV- IgA/IgG, EBV-NA, EBV-EA	ACV, PSL, Acetyl salicylic acid	20/100 (OD)
Hershberger (2003)^5^	0/M	Unilateral	XLP	Histological (retinal biopsy) In situ hybridization	NA	NA
Gallego-Pinazo (2008)^6^	5/M	Bilateral	NA	Serological EBV-IgM	ACV, Acetyl salicylic acid	20/40 (OU)
Hamam (2012)^7^	46/M	Unilateral	Chronic uveitis (Corticosteroids, MTX, CYA)	EBV-DNA 5.0 × 10^3^ copies/ml in VF	VACV	6/30 (OS)
Schaal (2014)^8^	55/F	Unilateral	Rheumatoid arthritis (Methyl-PSL, IFX)	EBV-DNA 2.4 × 10^3^ copies/ml in VF	GCV	NLP (OS)
Oe (2016)^9^	80/F	Bilateral	Rheumatoid arthritis (MTX)	EBV-DNA 2.1 × 10^5^ copies/ml in AH 5.0 × 10^6^ copies/ml in VF	GCV, VACV, PSL	LP (OD) 20/25 (OS)
Chan (2018)^10^	83/F	Unilateral	Hypertension, dyslipidemia	EBV-DNA 5.2 × 10^3^ copies/ml in AH 8.4 × 10^3^ copies/ml in VF	VACV	LP (OD)
Mashima (2019)^11^	83/F	Unilateral	Interstitial pneumonia, chronic pyelonephritis (MTX)	EBV-DNA <1.0 × 10^2^ copies/ml in serum 1.2 × 10^6^ copies/ml in AH 2.1 × 10^6^ copies/ml in VF	GCV, ACV (Intravitreal MTX)	20/125 (OS)
Current case	80/F	Unilateral	Rheumatoid arthritis (PSL, MTX)	EBV-DNA 3.5 × 10^2^ copies/ml in serum 4.2 × 10^6^ copies/ml in AH 1.2 × 10^8^ copies/ml in VF	ACV, FOS (Intravitreal GCV)	6/60 (OS)

XLP = X-linked lymphoproliferative disease, NA = not available, OD = right eye, OS = left eye, OU = both eyes, AH = aqueous humor, VF = vitreous fluid, NLP = no light perception, LP = light perception, EBV-NA = EBV nuclear antigen, EBV-EA = EBV early antigen, PSL = prednisolone, MTX = methotrexate, CYA = cyclosporine, IFX = infliximab, ACV = acyclovir, VACV = valacyclovir, GCV = ganciclovir, FOS = foscalnet

Among the series of EBV-ARN cases, more than half involved immunosuppressive states that were due to the underlying diseases or the use of immunosuppressants; however, immunocompetent patients are also involved.^4^^,^^6^^,^^10^ Typically, HSV-ARN and VZV-ARN develop in immunocompetent patients, whereas it is rarely seen in immunocompromised patients.^16^ In the present case, 5 mg/day of prednisolone and 2 mg/week of methotrexate were given for the treatment of rheumatoid arthritis at the onset; however, these doses were so small that the patient was not considered to be in a severe immunosuppressive state. However, it was known that methotrexate activated the release of infectious EBV from latently infected cells,^17^ and the mild immunosuppressive condition, such as the small amounts of oral methotrexate, may have been related to the onset of EBV-ARN.

When EBV is detected in the eye, its pathogenic role needs to be considered. The interpretation of positive results of EBV detected by qualitative PCR is controversial, because EBV can be detected in 20% of the ocular tissues from normal cadavers.^18^ The serological result is even more unconvincing. In some previous cases, diagnoses were based on EBV serology^4^^,^^6^; however, most healthy individuals within the population are EBV IgG-positive, accounting for 83–97% in Japan.^19^ In parallel with advances in molecular methods, quantitative PCR assay has become more widely available for clinical use. The vitreous quantitative PCR has been used for diagnosing EBV-related necrotizing retinitis in recent cases.^7^, ^8^, ^9^, ^10^, ^11^ Qualitative PCR on ocular fluid and/or blood serum alone is not sufficient to establish a conclusive diagnosis of an intraocular infection of EBV. To determine whether EBV plays a significant role in cases of ARN, it is necessary to compare the copy numbers of EBV-DNA in serum and vitreous fluid using the quantitative PCR assay.^20^ In this case, the copy numbers of EBV-DNA in the aqueous humor (4.2 × 10^6^ copies/ml) and vitreous humor (1.2 × 10^8^ copies/ml) were higher than that in serum (3.5 × 10^2^ copies/ml). From these results, we were confident about diagnosing this case with EBV-ARN.

EBV is a DNA virus of the herpesvirus family, as are HSV and VZV. EBV is known to cause infectious mononucleosis and is also associated with Burkitt's lymphoma and nasopharyngeal carcinoma. The virus exhibits affinity to B lymphocytes and nasopharyngeal epithelial cells.^21^ The antiviral drugs that are frequently used for the initial treatment of EBV infections have been acyclovir or ganciclovir,^22^ and these are also used with the EBV-related necrotizing retinitis cases.^4^^,^^6^, ^7^, ^8^, ^9^, ^10^, ^11^ The mechanism of action of acyclovir and ganciclovir is dependent on the formation of the nucleoside triphosphate, which is a strong inhibitor of the viral DNA polymerase. Acyclovir and ganciclovir must be monophosphorylated by virally encoded enzymes to be converted into nucleotides and incorporated into viral DNA for the formation of the nucleoside triphosphate.^15^ The herpes viruses develop resistance to acyclovir and ganciclovir when they have mutations in their virally encoded enzymes. It has also been suggested that EBV can acquire resistance to acyclovir and/or ganciclovir.^23^ In contrast, foscarnet inhibits the viral DNA polymerase by acting as an organic analogue of an inorganic pyrophosphate that directly inhibits the pyrophosphate binding sites on viral DNA polymerases, and it is reported that foscarnet can be effective in acyclovir-resistant HSV^15^ and even in EBV infection.^10^ In the present case, the patient was successfully treated with foscarnet.

## 4 Conclusions

We experienced a case of EBV-ARN that was resistant to systemic acyclovir and topical ganciclovir and responded effectively to systemic foscarnet. Although the clinical management remains challenging in this disease, foscarnet is considered to be one of the candidate drugs for EBV infections in the eye.

## Patient consent

Written informed consent was obtained from the patient for publication of this case report and any accompanying images.

## Funding

No funding or grant support.

## Intellectual property

We confirm that we have given due consideration to the protection of intellectual property associated with this work and that there are no impediments to publication, including the timing of publication, with respect to intellectual property. In so doing we confirm that we have followed the regulations of our institutions concerning intellectual property.

## Research ethics

We further confirm that any aspect of the work covered in this manuscript that has involved human patients has been conducted with the ethical approval of all relevant bodies and that such approvals are acknowledged within the manuscript.

IRB approval was obtained (required for studies and series of 3 or more cases).

Written consent to publish potentially identifying information, such as details or the case and photographs, was obtained from the patient(s) or their legal guardian(s).

## Authorship

The International Committee of Medical Journal Editors (ICMJE) recommends that authorship be based on the following four criteria:

Substantial contributions to the conception or design of the work; or the acquisition, analysis, or interpretation of data for the work; AND, Drafting the work or revising it critically for important intellectual content; AND, Final approval of the version to be published; AND, Agreement to be accountable for all aspects of the work in ensuring that questions related to the accuracy or integrity of any part of the work are appropriately investigated and resolved.

## Declaration of competing interest

The authors declare that they have no competing interests.
